# TAVR for All? The Surgical Perspective

**DOI:** 10.3390/jcdd9070223

**Published:** 2022-07-12

**Authors:** Xiling Zhang, Thomas Puehler, Derk Frank, Janarthanan Sathananthan, Stephanie Sellers, David Meier, Marcus Both, Philipp Blanke, Hatim Seoudy, Mohammed Saad, Oliver J. Müller, Lars Sondergaard, Georg Lutter

**Affiliations:** 1 Department of Cardiovascular Surgery, University Medical Center Schleswig-Holstein, Campus Kiel, 24105 Kiel, Germany; zhang_xiling@outlook.com (X.Z.); thomas.puehler@uksh.de (T.P.); 2 German Centre for Cardiovascular Research (DZHK), Partner Site Hamburg/Kiel/Lübeck, 24105 Kiel, Germany; 3 Department of Internal Medicine III (Cardiology, Angiology, and Critical Care), University Medical Center Schleswig-Holstein, Campus Kiel, 24105 Kiel, Germany; derk.frank@uksh.de (D.F.); hatim.seoudy@uksh.de (H.S.); mohammed.saad@uksh.de (M.S.); oliver.mueller@uksh.de (O.J.M.); 4 Centre for Heart Lung Innovation & Providence Research, Vancouver, BC V6Z 1Y6, Canada; jsathananthan@providencehealth.bc.ca (J.S.); ssellers@providencehealth.bc.ca (S.S.); dmeier@providencehealth.bc.ca (D.M.); 5 Centre for Cardiovascular Innovation, St Paul’s and Vancouver General Hospital, Vancouver, BC V6Z 1Y6, Canada; 6 Centre for Heart Valve Innovation, St. Paul’s Hospital, University of British Columbia, Vancouver, BC V6Z 1Y6, Canada; 7 Cardiovascular Translational Laboratory, Centre for Heart Lung Innovation, St Paul’s Hospital, Vancouver, BC V6Z 1Y6, Canada; 8 Department of Radiology and Neuroradiology, University Medical Center Schleswig-Holstein, Campus Kiel, 24105 Kiel, Germany; mboth@rad.uni-kiel.de; 9 Department of Radiology, St. Paul’s Hospital, University of British Columbia, Vancouver, BC V6E 1M7, Canada; phil.blanke@gmail.com; 10 Rigshospitalet, Copenhagen University Hospital, 2100 Copenhagen, Denmark; lars.soendergaard.01@regionh.dk

**Keywords:** aortic valve stenosis, transcatheter aortic valve replacement (TAVR), transcatheter aortic valve implantation (TAVI), surgical aortic valve replacement (SAVR), low risk, intermediate risk, high risk

## Abstract

In spite of the noninferiority of transcatheter aortic valve replacement (TAVR) in high- and intermediate-risk patients, there are still obstacles that need to be overcome before the procedure is further expanded and clinically integrated. The lack of evidence on the long-term durability of the bioprostheses used for TAVR remains of particular concern. In addition, surgery may be preferred over TAVR in patients with bicuspid aortic valve (BAV) or with concomitant pathologies such as other valve diseases (mitral regurgitation/tricuspid regurgitation), aortopathy, and coronary artery disease. In this review, we discuss and summarize relevant data from clinical trials, current trends, and remaining obstacles, and provide our perspective on the indications for the expansion of TAVR.

## 1. Introduction

As a result of a degenerative process, aortic valve stenosis (AS) is among the most common valvular diseases in developed countries [1,2], with an increasing incidence with increasing age, approximately 2.0% for those 65 years of age and 4.0% for those 85 years of age [3]. In patients over the age of 75, the incidence of persistent perioperative complications and mortality after surgical aortic valve replacement (SAVR) may be high, and other factors, such as significant comorbidities, female sex, frailty, New York Heart Association (NYHA) classification, or left ventricular dysfunction, may exacerbate the condition and significantly increase mortality [4,5].

**Figure 1:**
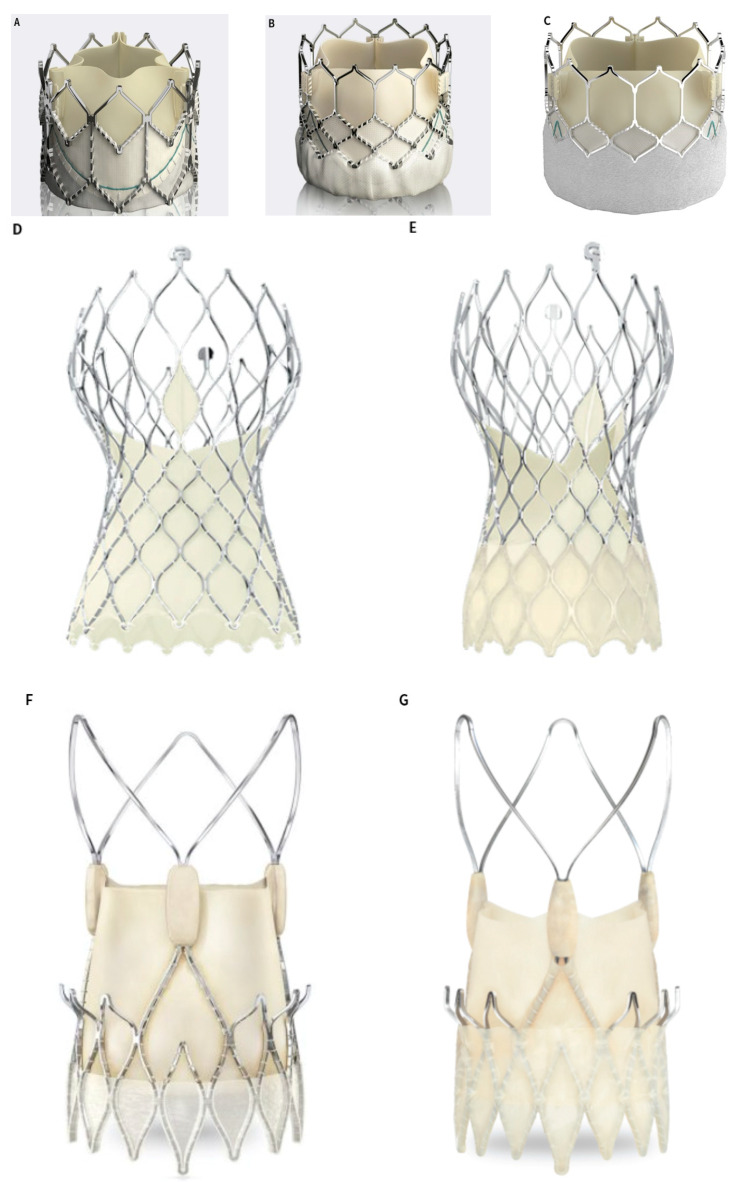

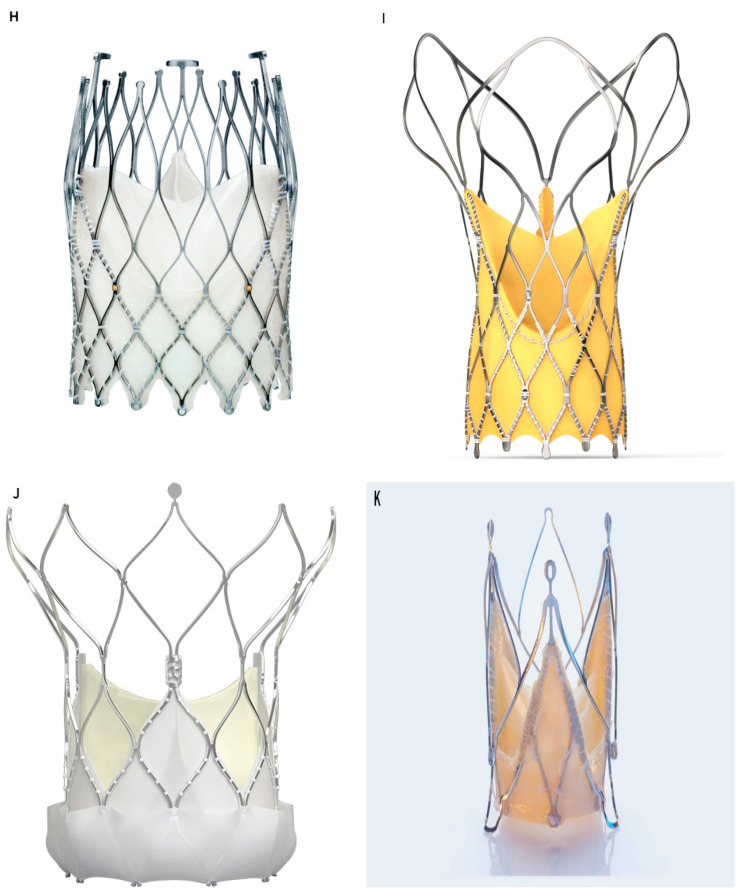
(**A**–**C**) The SAPIEN XT, SAPIEN 3 and SAPIEN 3 Ultra. Image courtesy of Edwards Lifesciences; (**D**,**E**) the Evolut R and Evolut R pro. Image courtesy of Medtronic; (**F**,**G**) the ACURATE neo and ACURATE neo2. Image courtesy of Boston Scientific; (**H**) the ALLEGRA. Image courtesy of NEW VALVE TECHNOLOGY; (**I**) the Hydra. Image courtesy of SMT; (**J**) the Navitor device, Image courtesy of Abbott; (**K**) the Jena valve, Image courtesy of JenaValve Technology.

More than one-third of AS patients are considered to be high-risk and are, therefore, deemed to be not eligible for SAVR. Due to this, transcatheter aortic valve replacement (TAVR) was initially developed as a procedure for inoperable patients. Since the first TAVR in 2002, these procedures have grown and exceeded the number of SAVR procedures performed in Germany [6,7,8]. TAVR has been shown to be noninferior to SAVR in a number of prospective randomized trials [9,10]. Figure 1 shows the currently available CE-marked transcatheter heart valves (THV).

**Figure 2:**
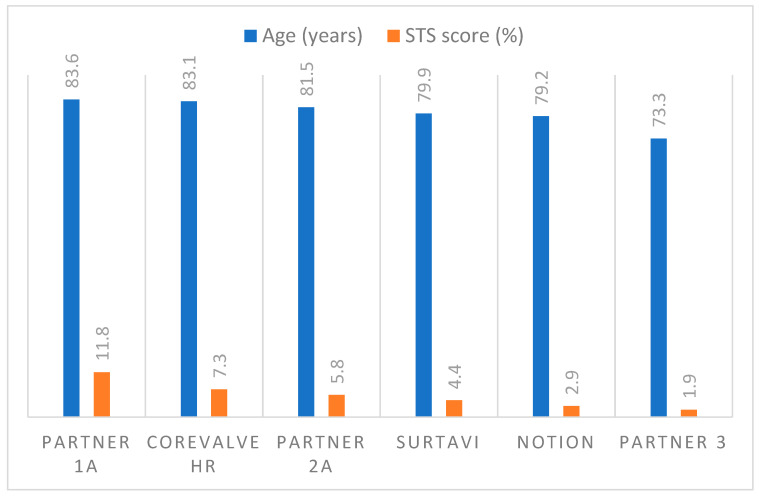
Studies on TAVR versus SAVR in patients at different surgical risks and of similar age. The progressive decrease in age and STS scores of patients implanted with TAVI.STS: Society of Thoracic Surgeons.

Additionally, it has been demonstrated that TAVR is noninferior in intermediate-risk patients [11,12,13]. Research is currently being conducted to determine whether TAVR can be extended to patients with low risk and younger ages (Figure 2). In this review, we summarize and discuss essential data from clinical trials, current trends, and remaining challenges, as well as discuss our perspectives on the expansion of TAVR.

## 2. TAVR for High- and Intermediate-Risk Patients

The indications for TAVR have evolved in recent years following the publication of results from multicenter randomized controlled trials.

The prognosis of conservative treatment in patients with inoperable aortic stenosis is inferior. Observational studies in these patients confirmed the safety and effectiveness of TAVR. Researchers concluded from the randomized controlled Placement of Transcatheter Aortic Valves (PARTNER) Trial 1B completed in 2010 that, compared with the conservative treatment group, TAVR with the balloon-expandable Edwards SAPIEN valve significantly reduced the 1-year mortality of inoperable AS patients (30.7% vs. 50.7%, *HR* = 0.55, 95% *CI*: 0.40–0.74, *p* < 0.001), and effectively improved cardiac function [14]; the five-year follow-up results showed that all-cause and cardiovascular mortality in the TAVR treatment group significantly decreased [15]. Similarly, the 2014 Core Valve Extreme Risk Pivotal trial demonstrated good efficacy and safety of TAVR in the treatment of extreme-risk patients [16] and extended findings of self-expanding valves for TAVR in high-risk patients. Based on these randomized controlled studies and large samples of observational studies [17,18], the American College of Cardiology/American Heart Association (ACC/AHA) guidelines for the management of valvular disease (2014) recommend TAVR for patients with severe AS who are inoperable and expect to live for more than 12 months (I/B) [19].

In the 2011 randomized controlled trial PARTNER 1A, the efficacy and safety of TAVR and SAVR were compared in patients with high-risk severe AS treated with the Edwards balloon-expandable SAPIEN valve. The study reported that, on average, the 1-year mortality rate of the two groups was not significantly different (24.2% vs. 26.8%, *p* = 0.44); the 5-year follow-up results indicated that, on average, the long-term effects of TAVR and valve durability were not inferior to SAVR [9,20]. Further, a study of the self-expanding CoreValve for patients with increased risk reported that the 1-year all-cause death rate in the TAVR group was significantly lower than that in the SAVR group (14.2% vs. 19.1%, *p* = 0.04) [10], and the 2-year all-cause death rate was also significantly lower than that in the SAVR group (22.2% vs. 28.6%, *p* = 0.04) [21].

Thus, the 2020 ACC/AHA valvular disease management guidelines recommended TAVR as the preferred treatment for high-risk and severe AS patients (>65 years) (I/A) and established a cardiovascular disease diagnosis and treatment team to assess a patient’s comorbidities and anatomical characteristics [1]. The 2020 European Society Of Cardiology (ESC) guidelines are slightly more restrictive: TAVR is recommended in older patients (≥75 years) or in those who are at high risk (STS-PROM/EuroSCORE II > 8%) or unsuitable for surgery (I/A) [22]. Notably, however, some research teams have presented conflicting study results. Armoiry et al. [23] used real-world evidence to compare 5-year clinical outcomes and direct costs between TAVR and SAVR. The results showed a higher risk of death at 1 year with TAVR than that with SAVR (16.8% vs. 12.8%, *HR* = 1.33; 95% *CI*: 1.02–1.72) and for up to 5 years (52.4% vs. 37.2%, *HR* = 1.56; 95% *CI*: 1.33–1.84). At 5 years, the risk of stroke, myocardial infarction, and pacemaker implantation were also increased after TAVR. Furthermore, the cost of hospitalization is also higher than that of SAVR (EUR 69,083 vs. EUR 55,687).

Despite some ongoing debate, TAVR has shown noninferior efficacy and safety compared with SAVR in severe AS patients at high and extreme risks, and as such, its use in severe AS patients at intermediate risk has received attention and prompted increasing clinical integration and evolution of trials; in the PARTNER 2A study, a randomized controlled trial presented in 2016, 2032 surgical intermediate-risk patients (STS score of 5.8%) were randomized to TAVR using the Edwards SAPIEN XT or SAVR. The 2-year follow-up showed no statistically significant difference between the two groups in the primary composite endpoint (all-cause death or disabling stroke) (19.3% vs. 21.1%, *HR* = 0.89, 95% *CI*: 0.73–1.09, *p* = 0.25), and in the subgroup analysis. TAVR via the femoral artery approach was found to be more effective than SAVR in reducing the incidence of the above endpoint events (*HR* = 0.79, 95% *CI*: 0.62–1.00, *p* = 0.05) [13]. SURTAVI trial (2017) evaluated the use of the CoreValve and Evolut R self-expanding valves in intermediate-risk patients and found that (1) the 2-year primary composite endpoint (all-cause death or disabling stroke) in the TAVR group was not higher than that in the SAVR group (12.6% vs. 14.0%, 95% *CI*: −5.2–2.3%; the posterior probability of noninferiority, >0.999), the transvalvular gradient (TG) in the TAVR group was lower than that in the SAVR group at 2 years, and the effective orifice area (EOA) was greater; (2) TAVR-group patients had a higher incidence of paravalvular leakage (PVL) and permanent pacemaker implantation (PPI) than SAVR-group patients, whereas SAVR-group patients had a higher incidence of postoperative acute kidney injury and new-onset or exacerbated atrial fibrillation (AF) [11].

With reference to the above evidence, the 2021 ESC guidelines recommended that TAVR may be the preferred option for patients with severe AS at intermediate surgical risk, when the transfemoral approach is feasible (I/B) [22]. TAVR may also be recommended as a treatment option for patients with severe AS at intermediate surgical risk by the guidelines (IIa/B-R) [24]. There is, however, an emphasis on the importance of determining the most appropriate treatment modality based on an individual risk assessment of the patient.

## 3. TAVI Transition to Low-Risk Patients

The most common complications of TAVR, including PVL, PPI, patient prosthetic mismatch, and valve durability, are arguably the most significant difficulties to address with this technology when considering its application to low-risk and young patients. According to current guidelines, SAVR remains the preferred treatment for symptomatic, low-risk severe AS [24,25]. In recent years, with improvements in TAVR surgical instruments, the optimization of procedures and procedural planning, as well as the reduction in complication rates, TAVR has become a research topic of increasing importance in the treatment of low-risk AS patients.

The NOTION study, completed in 2015, compared the effectiveness and safety of TAVR and SAVR in patients at lower risk (STS risk score of 3%, with 81.8% of patients having an STS risk score < 4%). A total of 280 patients were enrolled in the study, and the results of the 1-year follow-up indicated the following: (1) There were no statistically significant differences between the TAVR and SAVR groups in terms of the composite endpoint (all-cause death, stroke, and myocardial infarction, 13.1% vs. 16.1%, *p* = 0.43), and the incidence of all-cause death (4.9% vs. 7.5%, *p* = 0.38) and stroke (2.9% vs. 4.6%, *p* = 0.44) were not statistically significant. (2) The incidences of PPI (38.0% vs. 2.4%, *p* < 0.001) and aortic regurgitation (AR) (15.7% vs. 0.9%, *p* < 0.001) were significantly higher in the TAVR group than those in the SAVR group, whereas the incidences of postoperative major bleeding, acute kidney injury, and new onset of AF were significantly higher in the SAVR group, but major vascular complications did not differ between the two groups [12].

The NOTION study published 5-year follow-up results in 2019 and reported that the difference in the composite endpoint events between the TAVR and SAVR groups at 5 years remained statistically insignificant (38.0% vs. 36.3%, *p* = 0.86); it also noted that the rates of all-cause death (27.6% vs. 28.9%, *p* = 0.75), stroke (9.0% vs. 7.4%, *p* = 0.65), and myocardial infarction (7.7% vs. 7.4%, *p* = 0.96) were also not statistically significant between the two groups. Ultrasonography demonstrated lower TG in the TAVR group, compared with the SAVR group (8.2 mmHg vs. 13.7 mmHg, *p* < 0.001) and greater EOA (1.7 cm^2^ vs. 1.2 cm^2^, *p* < 0.001). However, the incidences of PPI (43.7% vs. 8.7%, *p* < 0.001) and AR (7.1% vs. 0%, *p* < 0.001) were higher in the TAVR group than those in the SAVR group [26].

In PARTNER 3, 1000 low-risk patients with severe AS (STS risk score of 1.9%) were randomly allocated to receive TAVR (using SAPIEN 3 THV with transfemoral access) or SAVR, respectively. The results of the 1-year follow-up showed the following: (1) The incidence of the composite endpoint event (all-cause death, stroke, or rehospitalization) was lower in the TAVR group than that in the SAVR group (8.5% vs. 15.1%, *HR* = 0.54, 95% *CI*: 0.37–0.79), with all-cause death rates of 1.0% and 2.5% (*HR* = 0.41, 95% *CI*: 0.14–1.17), stroke rates of 1.2% and 3.2% (*HR* = 0.38, 95% *CI*: 0.15–1.00), and rehospitalization rates of 7.3% and 11.0% (*HR* = 0.65, 95% *CI*: 0.42–1.00), respectively. (2) In terms of vascular complications, PPI, and moderate-to-severe PVL, the difference between TAVR and SAVR was not statistically significant [27]. The 2-year follow-up results of the concurrently published Medtronic Low-Risk TAVI trial showed that the incidence of the composite endpoint event (death or disabling stroke) was not statistically significant in the TAVR group, compared with that in the SAVR group (5.3% vs. 6.7%, posterior probability of noninferiority, >0.999), and the incidence of 30-day stroke, bleeding, acute kidney injury, and new-onset AF were lower in the TAVR group than those in the SAVR group. However, the incidence of moderate-to-severe PVL and PPI was higher in the TAVR group. At 1-year follow-up, echocardiography showed that patients in the TAVR group had lower TG than those in the SAVR group [28].

In the Evolut Low-Risk trial, 1403 patients underwent TAVR or surgical procedure (725 in the TAVR group and 678 in the surgery group). The patients’ mean age was 74 years. At 30 days, the TAVR group performed a lower incidence of disabling stroke (0.5% vs. 1.7%), bleeding complications (2.4% vs. 7.5%), acute kidney injury (0.9% vs. 2.8%), and AF (7.7% vs. 35.4%) with a higher incidence of moderate or severe AR (3.5% vs. 0.5%) and pacemaker implantations (17.4% vs. 6.1%), compared with the surgery group. At 12 months, patients in the TAVR group had lower aortic-valve gradients than those in the surgery group (8.6 mm Hg vs. 11.2 mm Hg) and larger EOA (2.3 cm^2^ vs. 2.0 cm^2^). The results of the 24-month follow-up showed that the primary endpoint (composite of death or disabling stroke) was 5.3% in the TAVR group and 6.7% in the surgery group (difference, −1.4 percentage points; 95% Bayesian credible interval for difference, −4.9 to 2.1; posterior probability of noninferiority > 0.999).

Although the above clinical trials demonstrate the efficacy and safety of TAVR in low-risk patients with AS, long-term follow-up data are still lacking. In addition, it is important to evaluate the risk of surgical death objectively. In fact, all widely used stratification tools have significant limitations in predicting surgical mortality [29,30]. These risk models did not include certain factors, such as a porcelain aorta, malignancy, and neurological impairment. Notably, the average age of patients from these low-risk trials was over 70 years. There are still unanswered questions when considering TAVR in younger patients (<70 years), especially since the sequence of multiple procedures over the patients’ lifetime is still unknown.

Schaefer et al. [31] used a real-world low-risk patient cohort to determine the different outcomes between SAVR and TAVR. The results indicated that the 3- and 5-year follow-ups showed increased mortality in the matched TAVR cohort, compared with the SAVR cohort (5-year survival 9.99% vs. 32.96%, *p* = 0.013).

Thus, for low-risk patients, especially those in younger age groups, with a need for optimal long-term results, the role of TAVR still requires careful individualized evaluation and shared decision making with patients, including uncertainty about long-term outcomes, as well as the potential for redo-TAVR.

## 4. TAVR for All?—Current Challenges: Bicuspid Aortic Valve

**Figure 3:**
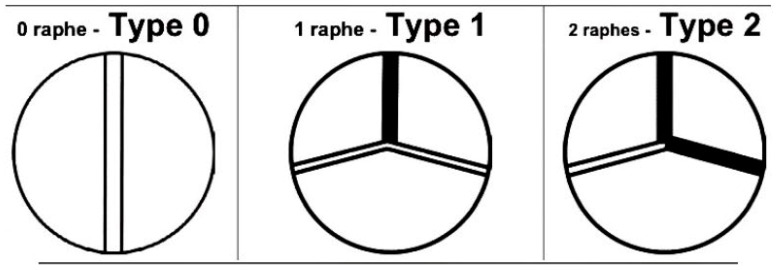
The Sievers classification for a BAV. The black line in schematic drawings represents a raphe, which is the nonseparated or conjoint segment of two underdeveloped cusps extending into the commissural area [35].

Bicuspid aortic valves (BAVs) are among the most common congenital malformations, affecting approximately 2% of the population [32]. They have a distinct anatomical structure: The shape of the valve leaflets is asymmetric, and the sinus opening is elliptical in shape. Thus, blood flow at the valve opening is blocked, flow velocity increases, and a vortex develops, resulting in thickening and asymmetric calcification of the valve. At the same time, due to its genetic mechanism and abnormalities, blood flow impinges on the aortic wall, making it more vulnerable to aortic wall lesions, such as ascending aorta dilatation and aneurysms. BAVs are prone to accelerate aortic valve calcification and require invasive treatment at a younger age than the tricuspid aortic valve (TAV) [33,34]. However, BAV patients have a long life expectancy and, therefore, will require longer durability of their valve replacement compared with typical AS patients with TAV. Thus, life-long planning for the valve replacement needs to be considered, and TAVR should only be performed on them if there is a high rate of device success, a low rate of reoperation, and long-term prosthesis durability. Figure 3 demonstrates the Sievers classification of BAV.

TAVR has become increasingly popular among patients with intermediate- and high-risk severe AS in recent years. Nevertheless, all major clinical trials have excluded patients with BAV.

Current guidelines list it as a relative contraindication based on the following factors: (1) annular shape—the oval annulus may affect the position of the implanted valve and the sealing beneath the implant; (2) calcification—asymmetric and severe leaflet calcification may affect valve expansion and postimplantation hemodynamics (e.g., higher transvalvular pressure gradient and PVL); (3) aortic structure—concomitant aortic disease increases the risk of aortic dissection and rupture during balloon dilation; (4) long-term prognosis—inadequate expansion and elliptical shape of the valve leaflets may affect the durability of the implanted valve.

Studies have reported the safety and efficacy of TAVR in patients with BAV. In 2017, SANNINO et al. [36] retrospectively analyzed the safety and efficacy of TAVR in 77 patients with BAV and 735 patients with TAV. The results indicated that there was no statistically significant difference in the success rates of valve implantation between BAV and TAV patients (98.7% vs. 99.1%, *p* = 0.556), as well as no differences in mortality, PPI, and moderate-to-severe PVL incidences at 30 days and 1 year after the procedure. During postoperative ultrasound evaluation, there was no difference in TG and peak flow velocity between the two groups. However, EOA was slightly larger in the BAV group than that in the TAV group (2.15 cm^2^ vs. 1.90 cm^2^, *p* = 0.007). In a matching analysis of 28 BAV patients and 84 TAV patients, KOCHMAN et al. [37] demonstrated that both groups of patients had similar rates of valve implantation success, aortic annulus rupture, and intraoperative conversion to SAVR. There was no statistically significant difference in 30-day and 1-year mortality or in TG, EOA, and rates of PVL. YOON et al. [38] compared the efficacy of TAVI in 546 BAV and 546 TAV patients through propensity score matching, and the results showed that the proportion of intraoperative conversion to SAVR in the BAV group was higher than that in the TAV group (2.0% vs. 0.2%, *p* = 0.006). The incidences of aortic root injury and moderate-to-severe PVL was higher than those of the TAV group.

However, in the subgroup analysis, it was found that the above differences between BAV patients implanted with new-generation valves (SAPIEN 3 and Lotus) were not statistically significant, compared with the TAV group. The follow-up results showed that there was no significant difference in the incidence of all-cause death and stroke between the two groups at 30 days and 2 years (17.2% vs. 19.1%, *p* = 0.28).

Two recent studies, LRT Bicuspid [39] and Evolut Low-Risk Bicuspid [40], both demonstrated excellent results at 30 days. One patient in the LRT Bicuspid study had moderate PVL at 30 days, and no deaths or disabling strokes were reported. In the Evolut Low-Risk Bicuspid study, the all-cause mortality rate and disabling stroke rate at 30 days were both 0.7%, and no patients had moderate or severe PVL. Several studies (BIVOLUT-X registry [41]: 5.3%; LRT Bicuspid study: 1.6%; and Evolut Low-Risk Bicuspid study: 4%) showed a higher rate of stroke than low-risk trials in patients with TAV (LRT: 0%; Evolut Low Risk: 3.4%). In a postmarket analysis from the STS/ACC TVT Registry, TAVR with balloon-expanded THV for the bicuspid valve was associated with an increased risk of stroke, compared with TAVR for the tricuspid valve (TAVR for bicuspid 2.5% vs. TAVR for tricuspid 1.6%; *p* = 0.02) [42]. These studies all suggest that the increased risk of stroke in patients with BAV is a problem that cannot be ignored.

The study by Majmundar et al. [43] suggested that, compared with SAVR, TAVR was associated with a lower in-hospital mortality (0.7% vs. 1.8%, *OR*: 0.35, 95% *CI*: 0.13–0.93; *p* = 0.035) and a similar rate of major adverse cardiovascular events at 30 days (1% vs. 1.5%, OR: 0.65, 95% *CI*: 0.27–1.58; *p* = 0.343) and at 6 months (4.2% vs. 4.9%, *HR*: 0.86, 95% *CI*: 0.44–1.69; *p* = 0.674) in the postmarket cohort. However, a long follow-up is still needed to compare the superiority and inferiority of TAVI and SAVR for BAV patients.

Recently, Yoon et al. [44] performed a computed tomography (CT) analysis of 1034 TAV patients and found that excessive leaflet calcification and moderate raphe calcification were significant predictors of all-cause death, PVL, and aortic root injury. In patients with both calcified raphe and excessive leaflet calcification, the incidence of aortic root injury was up to 4.5%, and the incidence of toxic-to-severe PVL was up to 6.5%, with All-cause mortality of 25.7%. In contrast, patients without the highest-risk phenotype showed excellent procedural outcomes. However, BAVs, particularly Sievers type 0 valves, were uncommon in this study. According to Husso et al. [45], BAVs of type 1 N-L and type 2 L-R/R-N morphologies have significantly higher incidences of mild-to-severe PVL (37.5% and 100%, respectively) than other types of BAVs.

Surgical outcomes are generally unaffected by aortic valve structure, and therefore, surgery should be preferred for patients with the highest-risk phenotype. Moreover, CT evaluation for BAV morphology may help identify high-risk and suitable patients. Therefore, further investigations into the BAV morphology in TAVI patients are necessary since the current findings suggest that these subtypes may be contraindicated for TAVR.

However, Patients with BAV often have associated thoracic aortic pathologies such as aortic root/ascending aortic dilatation and aneurysms. In patients with a BAV with indications for surgery and a diameter of aortic sinuses or ascending aorta ≥45 mm, the aortic sinuses and/or ascending aorta replacement is reasonable [1]. Current TAVR devices do not address the concomitant aortic pathology, and therefore, TAVR is limited to patients with isolated aortic disease.

## 5. Durability of Transcatheter Heart Valves (THVs)

**Figure 4:**
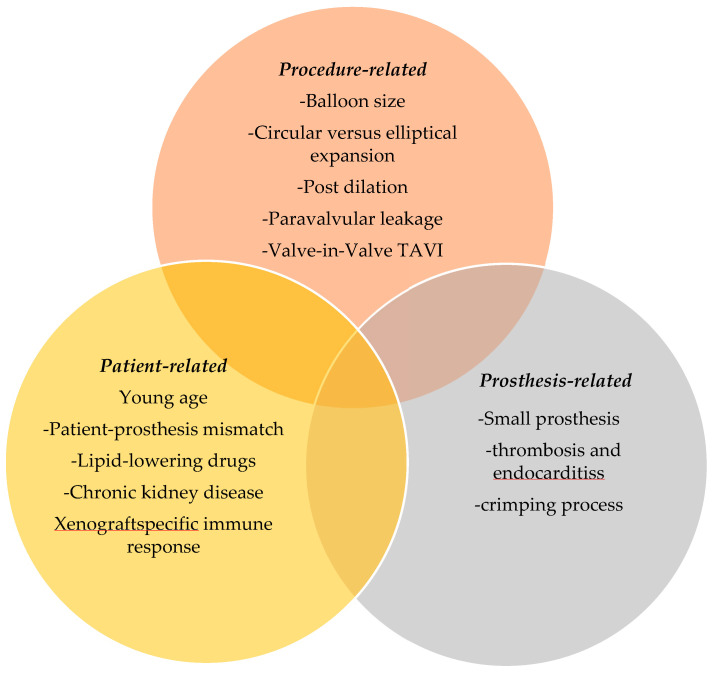
Patient-, prosthesis- and procedure-related factors involved in degeneration of bioprosthetic valves in stents.

Bioprosthetic valve dysfunction may result from structural or nonstructural causes. Structural valve degeneration (SVD) is characterized by fibrous calcification remodeling of the leaflets leading to tearing and rupture [46,47,48]. Nonstructural valve degeneration includes valve thrombosis, endocarditis, and PVL [49]. The mechanisms of SVD involve patient, prosthetic, and procedure factors (Figure 4).

A consensus paper published in 2017 provided the first standardized definition of SVD [49], which was divided into morphologic dysfunction (based on imaging findings of frame and leaflet function, integrity, and structure) and hemodynamic dysfunction (based on echocardiographic findings). The valve academic research consortium’s three criteria subdivide SVD into three stages—stage 1: morphological valve deterioration, stage 2: moderate hemodynamic valve deterioration, and stage 3: severe hemodynamic valve deterioration [50]. Moderate-to-severe hemodynamic SVD is defined as mean gradient >20 mmHg and/or mean gradient increase > 10 mmHg from three months postprocedure and/or new onset of more than mild aortic valve regurgitation or worsening from three months postprocedure.

The results of the first low-risk trial, the NOTION trial [51], showed better hemodynamic parameters with wider EOA (1.53 cm^2^ vs. 1.16 cm^2^, *p* = 0.002) and lower mean aortic gradient (9.9 mmHg vs. 14.7 mmHg, *p* = 0.001) in TAVR, compared with SAVR after 6 years of follow-up. The incidence of SVD was significantly lower in TAVR than in SAVR (4.8% vs. 24%, *p* < 0.001). However, the incidence of moderate-to-severe PVL was significantly higher in TAVR prosthesis than in SAVR (20.9% vs. 1.5%, *p* = 0.001). This may be due to the absence of an outer sealing skirt in first-generation devices and the fact that CT scans were not involved at that time to determine aortic annulus size.

To date, some data on the long-term durability of surgical bioprostheses are available. Thus, in one report, actuarial survival rates including early death were 78% ± 2%, 55% ± 2%, and 16% ± 2% at 5, 10, and 20 years of follow-up, respectively. The freedom rate from reoperation for prosthesis valve dysfunction in patients younger than 60 years averaged 98% ± 1%, 90% ± 3%, 60% ± 6%, and 30% ± 8% at 5, 10, 15, and 20 years follow-up, compared with 99% ± 0.3, 95% ± 1%, and 90% ± 3% at 5, 10, and 15 years follow-up in patients who between 60 and 70 years old, and 100% and 99% ± 0.5% at 5 and 10 years follow-up in patients older than 70 years [52]. On the other hand, for THVs, long-term data are only available for patients at high surgical risk [15,53] and those receiving first-generation devices [26], limiting the applicability of those findings to lower risk and younger patients.

Indeed, for low-risk patients with long life expectancy, the durability of THV is a critical issue. Additional long-term durability data are still needed to further define the role of TAVR in this population. Further, we must seek to understand the underlying mechanical and cellular mechanisms that drive bioprosthetic valve degeneration. Bench studies on valve durability and damage associated with procedural techniques and studies on biological causes of degeneration suggest a complex interplay between device host-response, mechanics and tissue durability, flow dynamics, and cellular mechanism of valve leaflet degradation and restriction. These biological mechanisms are supported by clinical imaging studies of the feature of TAVR degeneration [54,55,56,57,58,59]. Despite more than a decade of development, there is still no ideal prosthetic valve. Studies from the bench and the clinic will continue to inform the evolution of procedures and the development of new valve designs. However, physicians need to be careful when selecting new devices because some devices may not yet have the expected longevity. With the development of transcatheter technology, more attention now needs to be paid to the data related to the durability of bioprosthetic valves. Tissue engineering heart valve (TEHV) aims to create a valve graft with self-repair and remodeling capacity that may provide life-long durability. THEV may be a solution for next-generation TAVR devices to overcome the existing problems and maintain the advantages of the minimally invasive procedure. Helder et al. [60] compared cryopreserved decellularized aortic valve homograft with cryopreserved homograft and showed that freedom from reoperation after 10 years is higher for cryopreserved homograft than for cryopreserved decellularized aortic valve homograft (80% vs. 51%). Ureidopyrimidone-based tissue-engineered TAVR has been tested in acute sheep model and demonstrated good acute valve performance without regurgitation (*n* = 12) or with trace (*n* = 6) or mild (*n* = 2) regurgitation [61]. Although several challenges remain to be addressed, TEHV still has excellent potential in the future.

## 6. Risk of Valve-in-Valve TAVR and TAVR-in-SAVR Procedure

Current countermeasures for THV failure are mainly transcatheter valve-in-valve (ViV-TAVR) and SAVR. However, there is little evidence available from such studies. PARTNER trial showed that significant PVL was seen in 2.5% of patients who received ViV-TAVR, and the procedure demonstrated a high risk of morbidity and mortality [62]. Compared with redo SAVR, ViV-TAVR appears to have better short-term outcomes, even when performed in patients at increased surgical risk, and it is associated with less dialysis and PPI [63]. However, concerns about long-term outcomes, especially rehospitalization, persist [64].

The first report from the Redo-TAVR registry demonstrated that residual AR appeared to be more common, while residual valve gradient appeared to be more favorable than those observed in TAVR-in-SAVR [65,66,67,68]. Landes et al. [69] reported that the incidence of high residual gradients was 14.6% after ViV-TAVR and 21.5% after TAVR-in-SAVR (*p* = 0.095). The other report demonstrated that the rate of high residual gradients after TAVR-in-SAVR was 28% [70]. The larger internal diameter, the lack of sewing rings, and the greater expansiveness of the THV may account for these results. A bioprosthetic valve fractures procedure may ameliorate this situation in TAVR-in-SAVR patients. However, there are no available data on valve fractures. Overexpanded THV may reduce the residual gradient after TAVR [71].

One of the most serious complications of ViV-TAVR is acute coronary artery obstruction, and the incidence of acute coronary artery obstruction during and after ViV-TAVR is up to 2–3 fold higher than that after the first TAVR procedure [72]. Once it occurs, patients have a mortality rate of up to 40% within 30 days after the procedure [73]. In the case of redo-TAVR, the failed THV may form a “tube graft” in which the index THV leaflet will be jailed between two THV frames, forming a new skirt of tissue that flows from the failed THV to the top of the jailed leaflet [74], which may limit subsequent coronary access and flow. The entire leaflet of index THV may not be fully entrapped between the two THVs depending on the frame height and position of the second THV. The resulting neo-skirt height and degree of lobular overhang may affect THV performance, durability, and ability to access the coronary arteries [74]. In the case of the Evolut THV (Medtronic), redo TAVR with a shorter valve frame may have important technical implications for the neo-skirt height and leaflet overhang of the index Evolut THV. Access to the coronary artery through the Evolut THV frame may be more successful if the neo-skirt height is shorter, and the degree of leaflet overhang is greater. Conversely, expanding of index frame with an expandable balloon frame may result in greater expansion of the failed index Evolut THV, thereby limiting the space of the Valsalva sinus and increasing the risk of sinus sequestration and coronary artery obstruction. Akodad et al. [75] reported that placing SAPIEN 3 valve at a lower implant position in an index Evolut R valve reduced the height of the neo-skirt and had no significant compromise on the function of the SAPIEN 3 valve despite a higher degree of leaflet overhang. Integrating the patients’ anatomy on the basis of CT evaluation will assist in the preprocedural evaluation and procedure planning [76].

In the face of this complication, prophylactic stenting in potentially affected coronary arteries is a treatment option, but this technique has a higher rate of stent compression and thrombosis [77,78]. The BASILICA trial [79,80] proposed to lacerate the bioprosthetic or native aortic scallop immediately prior to placement of the prosthesis. Although this trial obtained 100% freedom from coronary obstruction, evidence is still needed via a prospective trial.

To date, there are no long-term studies available to assess the durability of such interventions. However, bench studies can provide guidance on these new procedures including assessment of different valve combinations and complication mitigation strategies [81,82,83]. With the expansion of the indication for TAVR in younger patients at low surgical risk, the number of TAVR is rapidly increasing, which will increase the number of ViV-TAVR procedures in the coming years. In this context, it is crucial to evaluate the prognosis of ViV-TAVR carefully and to compare ViV-TAVR with TAVR-in-SAVR. Additionally, and more importantly, determining the optimal sequence of interventions for patients is critical, especially for younger patients (<65 years) who may need to undergo multiple aortic valve interventions. TAVI first, followed by surgery with explant of the TAVI and implantation of the surgical aortic valve (SAV), followed by TAVI in SAV later on might be an option. However, surgery to explant THV shows higher risks and requires a surgeon with extensive experience. The “TAVR only” strategy seems to be less appropriate for younger patients because this approach carries a risk of explanting numerous calcified cusps and stent struts, which can pose high risks and challenges for surgeons performing SAVR later on. In addition, if the second THV degenerates prematurely, the patient may face very few options for a new THV [84]. Moreover, young patients often reject mechanical valves due to the need for anticoagulation. In the literature, there are some cases without anticoagulation for over 30 years without significant embolic events [85]. Mechanical valves without anticoagulation may become available in the future, which may revolutionize the treatment strategy for young patients.

## 7. Concomitant Cardiac Pathology

According to current guidelines [19,24,25], SAVR remains the treatment strategy of choice for patients with BAV with aortopathy >4.5 cm, patients with a multivessel disease with high Synergy of Percutaneous Coronary Intervention and Cardiac Surgery (SYNTAX) scores or left main coronary artery or patients with severe mitral valve disease and infected endocarditis requiring surgical removal of infected tissue.

## 8. Isolated Severe Aortic Valve Regurgitation

Conservative treatment of symptomatic severe AR is ineffective. SAVR remains the standard treatment for these patients. However, like severe AS, some patients have high surgical risks and high postoperative mortality, resulting in many patients losing the chance of surgery. Can such patients benefit from the TAVR procedure? Patients with isolated AR have less leaflet calcification and are often associated with dilation of the ascending aorta and aortic annulus. These factors may lead to difficult TAVR valve anchoring, inaccurate positioning and release, and a high incidence of moderate-to-severe PVL after surgery. Studies have shown that these patients have a higher incidence of moderate-to-severe PVL during TAVR procedures requiring two-valve implantation (valve-in-valve) [86].

SAWAYA et al. [87] reported the therapeutic effect of TAVR in the treatment of Isolated severe AR. The results showed that the device implantation success rate, early survival rate, and clinical effectiveness were 72%, 66%, and 61%, respectively. The success rate of device implantation (85% vs. 54%, *p* < 0.05) and clinical efficiency (75% vs. 46%, *p* < 0.05) were higher than those of the first-generation device, especially the incidence of moderate/severe PVL was significantly decreased (3% vs. 27%, *p* = 0.012). The safety and efficacy of the JenaValve Trilogy for the treatment of aortic regurgitation was recently published at EuroPCR 2022. All patients (45) achieved technical success, with no conversion to open surgery, no life-threatening bleeding, one patient with major vascular complications (2.2%), and nine patients required pacemakers (23%). At discharge, the patients’ mean aortic valve gradient was 4.04 ± 1.64 mmHg (9.85 ± 7.81 mmHg at baseline), and the mean aortic valve area was 2.62 ± 0.64 cm^2^ (2.07 ± 0.79 cm^2^ at baseline). Overall, 91% of patients were discharged with no or trace paravalvular regurgitation, and none had more than moderate regurgitation. It is believed that with the continuous accumulation of experience and the continuous improvement of the valve, the effect of TAVR in the treatment of such patients is expected to be further improved.

## 9. Mild PVL, Heart Block

Surgery can reduce the incidence of PVL by directly removing the severe calcification around the annulus. Although the overall incidence of PVL after TAVR has decreased with the use of newer generation devices, the incidence of mild PVL is consistently higher than that of SAVR. Mild PVL is associated with increased mortality in high-risk patients [17,88,89]. Despite the continuous improvement of implanted valve materials and design and operating techniques, conduction block is still one of the main complications after TAVR. The most common ones are left bundle branch block (LBBB) and complete atrioventricular block (AVB), with incidence rates ranging from 4% to 65% and 2% to 51%, respectively [90,91].

The mechanical compression of the atrioventricular node and conduction bundle by the implanted valve is an important reason for the occurrence of conduction block. Some patients can recover from conduction blocks after the procedure, and some patients need to implant a permanent pacemaker (PPM). The presence of preoperative right bundle branch block (RBBB), prolonged PR interval, deep valve entry into the left ventricular outflow tract, and large valve size are the main risk factors for postoperative implantation of a PPM in patients [92]. For patients with new-onset LBBB after TAVR that lasted for more than 48 h, AUFFRET et al. [91] suggested that the patients be divided into three types according to the QRS width and whether they were combined with first-degree AVB: (1) QRS < 160 ms, without first-degree AVB; (2) 130 ms < QRS < 160 ms, with first-degree AVB; (3) QRS ≥ 160 ms; it is also recommended that type 1 does not require implantation of a pacemaker, and types 2 and 3 should undergo electrophysiological examination or directly implant a PPM. Whether implanting a PPM affects patient outcomes requires more long-term follow-up studies to confirm.

## 10. Conclusions

TAVR is indeed a boon for those patients who cannot undergo surgery and those who are at intermediate to high risk. However, TAVR still comes with some unresolved issues, such as PVL, PPI, and the durability of THV is still not fully supported by long-term studies. TAVR may also not always be appropriate for some special populations, such as patients with BAV and patients with isolated severe aortic valve regurgitation.

Moreover, in younger patients, the use of mechanical valves may still be a better option, and care must be taken not to over-expand the indications for TAVR and generalize results in populations where its use has not been formally studied. Without caution, patients may reject open-heart surgery based on practical issues, such as faster recovery and less pain, but the heart team should be responsible for educating patients when their perceptions of open-heart surgery are inaccurate. The fact that open-heart surgery and extracorporeal circulation are safe should be used as a benchmark for judging evolving interventions. In the era of TAVR, each program should conduct regular internal reviews of surgical and transcatheter outcomes, the goal should be the lowest cumulative mortality, and as more data become available, the cardiac team should make reasonable decisions based on a thorough evaluation of the patient and the most recent data.

## Data Availability

Not applicable.

## Abbreviations

The American College of Cardiology/American Heart Association	ACC/AHA
Atrial fibrillation	AF
Aortic regurgitation	AR
Aortic valve stenosis	AS
Atrioventricular block	AVB
Bicuspid aortic valve	BAV
Confidence interval	CI
Computed tomography	CT
Effective orifice area	EOA
European Society of Cardiology	ESC
Hazard ratio	HR
Left bundle branch block	LBBB
New York Heart Association classification	NYHA classification
Permanent pacemaker	PPM
Permanent pacemaker implantation	PPI
Paravalvular leakage	PVL
Right bundle branch block	RBBB
Surgical aortic valve replacement	SAVR
Society of Thoracic Surgeons	STS
Structural valve degeneration	SVD
Synergy of Percutaneous Coronary Intervention and Cardiac Surgery	SYNTAX
Tricuspid aortic valve	TAV
Transcatheter aortic valve implantation	TAVI
Transcatheter aortic valve replacement	TAVR
Transcatheter heart valve	THV
Transvalvular gradient	TG
Transcatheter valve-in-valve	ViV-TAVR
